# Clinical Lecture in the U. S. Marine Hospital

**Published:** 1853-01

**Authors:** W. B. Herrick


*Clinical Lecture, in the U. S. Marine Hospital, by* W. B. Herrick, M. D.

The constituents of food, having been prepared in the alimentary canal, are taken up by the lacteals and mesenteric veins, and thus introduced into the circulation, as noticed in a previous lecture. We have seen that food may be divided, for the sake of convenience, into three classes,—the protein, or nitrogenized compounds; the oily and amylaceous, or carbonaceous compounds; and the mineral compounds. We shall find it convenient to keep in view this classification, in speaking of the blood itself, the changes to which it is subject, and the relation of these changes to the fluids and solids without the blood-vessels. In order to the proper performance of the process of secondary assimilation, it is evident that the different organic and inorganic substances should be supplied in the proper relative proportions. These substances, with the exception, perhaps, of some of the inorganic constituents, can reach the solids and extra vascular fluids only through the blood; the existence, therefore, in this fluid of all the constituents of tissue properly combined and organized, is necessary to health, and deficiency or excess of one or more of these will give rise to morbid phenomena.

We propose to examine each of these classes of substances separately,—their function in health, and their influence in disease.

The body, as a whole, may be divided into three classes of substances, corresponding to those which we have already noticed in the food and blood; and it is an interesting fact, that this chemical division corresponds with a physiological classification—for, as we shall see, each of these classes has a different function.

We may assume that the use, so far as structure is concerned, of the minerals is to build up the skeleton; of the protein compounds to build up the muscular and fibrous tissues; and of the carbonaceous, to be deposited in the form of fat, to enter into the composition of the nervous structures, and to subserve the purposes of respiration. But in passing to their ultimate destination, they each have other offices to perform, and some of each are found in all the structures of the body. In the economy of nature, the fewest possible agents are made use of for the accomplishment of any given result; and it is, no doubt, true that, from the first introduction of food into the system until its final removal by the excreting organs, it exerts a marked influence over the nutritive processes. Blood itself may be regarded, in one sense as a tissue, subject to growth and decay. The corpuscles and the fibrin have been regarded by physiologists as possessed of vital properties. In regard to fibrin, we think there may be room for doubt; but that the organic cells existing in the blood are as much living as muscular or nervous matter, we think cannot be denied. The precise manner of their formation, whether as so many separate and distinct cells, built up independently of other organic forms, or whether, as is contended by some recent observers, they are the escaped nuclei of the white corpuscles, is perhaps uncertain; but it is important only to know the conditions of their growth and decay.

An organic cell is a product of the three classes of substances which we have noticed—fat, albumen, and salts. If oil be intimately mixed with an alkali, it is divided into minute particles; and if to this emulsion albumen be added, and the temperature be kept at from 96° to 100° F., there is formed around these oil granules a layer of albumen and the inorganic compounds, the whole resembling, so far as we can see, the organic cells of living tissues. Among other experiments and observations on this subject, we would refer to those reported in the first No. of the present volume of the *North-Western Med. and Surg. Journal.* It is only claimed, in regard to those experiments, that the forces acting in the mixture to produce celluloid bodies, are probably acting in the animal to give form and structure to the cellular element; and, in order that we may have growth of the blood, or of any other tissue, we must have combined at least these three kinds of substances. It is probable that the character of the cell may be modified by the relative proportions of its constituents, and also by the number and arrangement of its different elements. For instance, iron seems to have an influence in producing the blood corpuscles, while alkalies and the alkaline earths probably have a greater influence in the manufacture, so to speak, of the softer solids of the body.

In cases, therefore, where we have reason to believe there is a deficiency of the blood globules, we may administer with confidence the ferruginous preparations. You have seen the effects of this kind of medication in those cases of anæmia after intermittents which have fallen under your observation in the wards of this hospital. In order that the condition of the blood may be improved, both as regards its nutritive quality and its globular element, we prescribe, in connection with iron in some of its forms, a generous mixed diet, guarding carefully against any vice of primary assimilation, and especially favoring the solution and consequent absorption of as much nutritious material as possible, by giving the mineral acids.

There may be a condition of the blood opposite to that which I have just noticed. The corpuscles and albumen, one or both of them, may be in excess, and our indication will be to reduce them by venesection. You will observe that the volume of the circulating fluid is not diminished, but that its quality only is affected by bleeding. We must discriminate, therefore, between mere fulness of the vessels, and a relative excess of some of the constituents of the blood.

We have spoken of the minerals as stimulants to organization; they may probably be also regarded as possessing a preserving influence over the already formed structures, as we shall see when we come to speak of the nutrition and destruction of the solids of the body. Changes are constantly taking plaee in the blood, dependent upon the substances introduced through the assimilative organs, from the extravascular tissues, and through the lungs; and the character of these changes will evidently be modified by any disturbance, relatively, of the amount of the substances introduced through these several channels. But perhaps none of them exert a more marked influence than oxygen. This agent seems to act in opposition to the mineral constituents, by furnishing the disorganizing force, reducing complex compounds to more simple ones, and thus favoring their elimination from the system. An excess or deficiency of this agent is, we believe, a prolific source of disease, and especially of alteration in the quality of the blood. It is probably true that a certain proportion of albumen is oxydized in the blood, and its products, urea and uric acid, eliminated by the kidneys. We think it not improbable that, as has been claimed by some observers, a diminution of the amount of oxygen may give rise to such a condition of the blood, that the albumen—unchanged, or, at least, but slightly modified—may pass into the renal tubuli, and thus we may have albuminuria. There are many facts which seem to sustain this idea. In nearly all conditions in which there is defective aeration of the blood, we find albumen in the urine, and this without any evidence of organic disease of the kidneys. This view is favored by the fact that, in diabetes, when there is an abundance of the hydro-carbonaceous element to divert the oxygen from the nitrogenized compounds, albumen is often found in the urine, and also that the urea in albuminous urine is below the normal standard.

There is another condition in which we may consider the hydro-carbonaceous element in excess—or the oxygen or blood globules, on which the amount of oxygen is dependent, deficient. We refer to a chylous condition of the blood, in which there is too much fat. We may evidently meet the indications in both of these conditions, by favoring the formation of blood corpuscles, and guarding against any vice in the performance of the function of respiration.

